# Exploring the differentiation potential of adipose tissue-derived mesenchymal stromal/stem cells and progenitor buccal epithelial cells into urothelial cells

**DOI:** 10.3389/fbioe.2025.1687541

**Published:** 2026-01-12

**Authors:** Monika Buhl, Arkadiusz Jundziłł, Paweł Dąbrowski, Kamil Szeliski, Marta Rasmus, Tomasz Kloskowski, Łukasz Kaźmierski, Tomasz Drewa, Marta Pokrywczyńska

**Affiliations:** 1 Chair of Urology and Andrology, Department of Regenerative Medicine, Collegium Medicum, Nicolaus Copernicus University, Bydgoszcz, Poland; 2 Department of Plastic, Reconstructive, and Esthetic Surgery, Collegium Medicum, Nicolaus Copernicus University, Bydgoszcz, Poland

**Keywords:** differentiation, urothelial cells, buccal epithelial cells, adipose tissue-derived mesenchymal stromal/stem cells, conditioned medium, progenitor cells, tissue engineering, urology

## Abstract

**Background:**

The intestinal wall is the most commonly used substitution material in reconstructive urology. However, this strategy is associated with many complications. Tissue engineering techniques can replace the intestinal wall with an *in vitro-created* urinary tract substitute. Biomaterial seeding with urothelial cells (UCs) would increase the success of the regeneration process. The problem in many patients requiring urinary reconstruction is the lack of autologous UCs. This study aimed to compare the differentiation potential of porcine mesenchymal adipose tissue-derived stromal/stem cells (AD-MSCs) and progenitor buccal epithelial cells (pBECs) toward UCs. UCs, AD-MSCs, and BECs were isolated from tissues harvested from domestic pigs. The conditioned medium was collected from UC cultures (UC-CM) between the first and third passage (n = 5). AD-MSC and BEC cultures were exposed to UC-CM for 7 and 14 days, respectively. UCs from the third passage (day 0) were used as a positive control. The differentiation effect of UC-CM was characterized by analysis of selected UC-typical markers at the mRNA and protein levels.

**Results:**

AD-MSCs subjected to urothelial differentiation changed their morphology from fibroblast-like to epithelium-like. In BECs, the differentiation factor increased the expression of urothelial markers, including *CK8*, *UPK1B*, and UPKIII.

**Conclusion:**

This study demonstrated that pBECs have a higher potential to differentiate toward urothelial-like cells than AD-MSCs. UC-CM is the source of factors inducing the differentiation of pBECs toward urothelial-like cells. Buccal epithelium can be used as a cell source for reconstructive urology applications.

## 1 Background

Radical cystectomy (RC) with urinary diversion using bowel segments is the gold standard for the treatment of muscle-invasive bladder cancer. However, using bowel segments is associated with many complications, including additional surgery on the intestine and different properties of the digestive and urinary tracts. Methods of tissue engineering create the possibility of replacing the intestinal wall with an *in vitro*-created tissue-engineered substitute and the construction of organs such as neo-bladders or neo-conduits from the patient’s own cells (Adamowicz et al., 2017; Pokrywczyńska et al., 2013). The tissue-engineered graft was first used in clinical practice for bladder reconstruction by Atala et al. (2006). The bladder wall substitute was constructed using a composite of PGA/C or BAM seeded with smooth muscle cells (SMCs) and urothelial cells (UCs) (Atala et al., 2006). Numerous studies have shown that the cell-seeded scaffold strategy is more effective than scaffolds without cells, increasing regeneration potential and reducing scar tissue formation. The number of implanted cells is a significant factor influencing the regeneration outcome (Adamowicz et al., 2017; Pokrywczyńska et al., 2013; Atala et al., 2006; Garriboli et al., 2014). Although there are urinary bladder and urinary tract diseases (invasive bladder cancer and interstitial cystitis with idiopathic detrusor overactivity), where autologous UCs and SMCs cannot be used (Adamowicz et al., 2017; Pokrywczyńska et al., 2013; Atala et al., 2006; Garriboli et al., 2014; Southgate et al., 2007).

The presence of autologous UCs for urinary tract reconstruction is critical because the UC layer serves as a tight and functional barrier. Seeding of UCs on the inner surface of the scaffold could prevent urine leakage into the deeper layers of the scaffold and avoid damage to the cells growing on its outer side (Drewa et al., 2004; Nagele et al., 2008; Pokrywczynska et al., 2014). Therefore, UC application in tissue engineering in urology can contribute to preventing complications, especially at the beginning of the regeneration process. Limited possibilities of using autologous UCs create a necessity for using external epithelial cell sources (Fan et al., 2017; Kang et al., 2014; Liu et al., 2009; Pokrywczynska et al., 2018; Shi et al., 2012; Turner, 2015; Zhang et al., 2013).

Differentiation methods of stem cells toward a UC-like phenotype provide hope for obtaining urothelial-like cells for application in reconstructive urology. However, to date, no effective, reproducible methods have been developed for obtaining cells with a urothelial phenotype from external epithelial cell sources (Fan et al., 2017; Kang et al., 2014; Liu et al., 2009; Pokrywczynska et al., 2018; Shi et al., 2012; Turner, 2015; Zhang et al., 2013).

The conditioned medium (CM) is obtained from the culture of the selected cell types. It is the source of factors that could differentiate cells, including extracellular vesicles, growth factors, and extracellular matrix proteins secreted into the culture medium by the cultivated cells (Staubach et al., 2021). Conditioned medium from UC cultures has been described in the literature as a potential source of factors that provide an environment to achieve the urothelial phenotype (Shi et al., 2012; Al.-kurdi et al., 2021; Tian et al., 2010; Zhang et al., 2014).

Bone marrow/adipose tissue-derived mesenchymal stromal/stem cells exhibit great potential for urinary tract regeneration (Liu et al., 2009; Shi et al., 2012; Turner, 2015; Zhang et al., 2013; Al.-kurdi et al., 2021; Tian et al., 2010; Zhang et al., 2014). The high availability, the separate panel of markers for identification, and their properties (especially the capability for multi-lineage differentiation) are the main reasons why AD-MSCs have been the most frequently used cells in trials of differentiation into cells with a urothelial-like phenotype (Viswanathan et al., 2019; Yoon and Ho, 2019; Fan et al., 2017; Kang et al., 2014; Liu et al., 2009; Pokrywczynska et al., 2018; Shi et al., 2012; Turner, 2015; Zhang et al., 2013). However, it should be emphasized that AD-MSCs develop from mesoderm (Sebo et al., 2018), while bladder UCs are derived from endoderm (Apodaca, 2004). Progenitor buccal epithelial cells (pBECs) isolated from the oral mucosal buccal epithelium also have the potential for regeneration of the urothelial layer. The similarities between UC and BEC phenotypes are presented in Table 1. Buccal tissue is derived from ectoderm, which has more features similar to the endoderm than the mesoderm (Sa et al., 2016; Squier and Kremer, 2001; Winning and Townsend, 2000; Jones and Klein, 2013), and pBECs have already been used in urology for urethroplasty (Hillary et al., 2014; Ławkowska et al., 2023; Smith, 2016). No attempts to generate urothelial-like cells from pBECs have been reported. In an earlier study by Hustler et al. (2018), key transcription factors (TFs) involved in determining the urothelial cell phenotype were identified by comparing TF expression profiles between human urothelial and buccal epithelial cells. Results showed that the overexpression of peroxisome proliferator-activated receptor gamma (PPARγ1) promotes a network of TFs implicated in driving urothelial-type differentiation (Hustler et al., 2018).

**TABLE 1:** Comparison of selected UC and oral epithelial cell markers.

Markers	Urothelial cells	Oral epithelial cells
Cytokeratin 1 (CK1)	Information not found	Expression in the keratinized oral epithelium (Wang et al., 2019)
Cytokeratin 2 (CK2)	Information not found	Expression in the keratinized oral epithelium (Winning and Townsend, 2000)
Cytokeratin 3 (CK3)	Information not found	Expression (Krishnan et al., 2010; Madhira et al., 2008)
Cytokeratin 4 (CK4)	Information not found	Expression in the non-keratinized oral epithelium (Winning and Townsend, 2000)
Cytokeratin 5 (CK5)	Expression by the basal layer (Hustler et al., 2018; Colopy et al., 2014; Jackson et al., 2019; Veranic and Jezernik, 2006; Wezel et al., 2014)	Expression by all layers of human oral epithelial cells (OECs) of buccal derivation (Squier and Kremer, 2001; Wang et al., 2019; Hustler et al., 2018)
Cytokeratin 6 (CK6)	Information not found	Expression in the keratinized oral epithelium (Winning and Townsend, 2000; Calenic et al., 2015)
Cytokeratin 7 (CK7)	Expression by all layers (Hustler et al., 2018; Wezel et al., 2014; Sun et al., 2014)	Absence of expression (Hustler et al., 2018)
Cytokeratin 8 (CK8)	Expression by all layers (Kang et al., 2014; Veranic and Jezernik, 2006; Kloskowski et al., 2014; Southgate et al., 1999)	Absence of expression (Hsueh et al., 2016)
Cytokeratin 10 (CK10)	Information not found	Expression in the keratinized oral epithelium (Wang et al., 2019)^,^ (Izumi et al., 2000)
Cytokeratin 12 (CK12)	Information not found	Absence of expression (Madhira et al., 2008)
Cytokeratin 13 (CK13)	Expression by the basal layer (Hustler et al., 2018)	Suprabasal expression (Squier and Kremer, 2001; Winning and Townsend, 2000; Wang et al., 2019; Hustler et al., 2018)
Cytokeratin 14 (CK14)	Absence of expression (Hustler et al., 2018)	Particularly intense expression by the basal layers of human OECs of buccal derivation (but not exclusive to this layer) (Wang et al., 2019; Hustler et al., 2018)
Cytokeratin 15 (CK15)	Expression by a basal layer (Tai et al., 2013)	Information not found
Cytokeratin 18 (CK18)	Expression by a layer of umbrella cells (Kloskowski et al., 2014; Mudge et al., 2005)	Information not found
Cytokeratin 19 (CK19)	Expression by all layers (Veranic and Jezernik, 2006)	Expression in the human and canine BECs. Absence of expression in the porcine BECs (Sa et al., 2016; Calenic et al., 2015)
Cytokeratin 20 (CK20)	Expression by the superficial cell layer (Hustler et al., 2018; Veranic and Jezernik, 2006)	Absence of expression (Hustler et al., 2018)
β1 integrin (ITGβ1)	Expression by all layers (Lieu et al., 2007)	High expression by the basal and suprabasal layer of oral epithelial cells of gingiva and hard palate derivation (Igarashi et al., 2008)
β4 integrin (ITGβ4)	Expression by the basal layer (Kullmann et al., 2017)	Expression by the OECs of buccal and gingiva derivation (Calenic et al., 2014)
P63	Expression by the stem cells (Wezel et al., 2014; Fishwick et al., 2017)	Expression in the OECs of buccal and gingiva derivation (Krishnan et al., 2010; Ghaemi et al., 2018)
P75 (neurotrophin receptor, NGFR)	Expression by the basal cell layer. More homogeneous and intensive expression in the case of the porcine urothelium compared to human urothelium (Wezel et al., 2014; Wezel and Southgate, 2011)	Expression by the stem cells of the basal layer in OECs of buccal and gingiva derivation (Jones and Klein, 2013; Nakamura et al., 2007)
Uroplakins 1a, 1b, 2 and 3 (UPKs 1a, 1b, 2, 3a, and 3b)	Expression by the layer of umbrella cells (Hustler et al., 2018)	Absence of expression (Hustler et al., 2018; Kullmann et al., 2017)

The aim of this study was to compare the differentiation potential of AD-MSCs and pBECs toward porcine UCs.

## 2 Materials and methods

### 2.1 Tissue collection

The source materials for cell isolation were urinary bladders (n = 60), subcutaneous adipose tissue of the abdominal wall (n = 20), and buccal mucosal tissues (n = 180) harvested from male domestic pigs (age ∼6 months), weighing on average 120 kg, during planned economic slaughter at a local slaughterhouse.

### 2.2 Histological and immunohistochemical staining

Hematoxylin and eosin (H&E) and immunohistochemical staining (IHC) were performed according to standard protocols to confirm the presence of urothelium, adipose tissue, and buccal oral epithelium in the examined tissue fragments. The primary monoclonal anti-CK7 (1:1500) and anti-UPKIII (1:32000) antibodies (Table 2) were used to test the expression of CK7 and UPKIII proteins. Microscopic evaluation was performed in three fields of view under a ×40 objective magnification (DMI6000B fluorescence microscope, Leica, Germany).

**TABLE 2:** Antibodies used for AD-MSC and pBEC characterization after trials of differentiation toward UCs.

Antibody	Used dilution	Identification data
Mouse monoclonal antibody against CK7	IF: 5 μg/ml FC: 2.5 μg/ml	Abcam, Cambridge, United Kingdom, Cat# ab9021
Goat anti-mouse IgG H&L (Alexa Fluor® 488)	IF: 4 μg/ml FC: 4 μg/ml	Abcam, Cambridge, United Kingdom, Cat# ab150113
Mouse IgG1, kappa monoclonal [15-6E10A7]-isotype control	FC: 2.5 μg/ml	Abcam, Cambridge, United Kingdom, Cat# ab170190
Rabbit monoclonal antibody against CK14	IF: 1.1 μg/ml	Abcam, Cambridge, United Kingdom, Cat# ab181595
Rabbit monoclonal antibody against CK14	FC: 3 μg/ml	Abcam, Cambridge, United Kingdom, Cat# ab236439
Donkey F (ab')2 Anti-Rabbit IgG H&L (Alexa Fluor® 488)	IF: 4 μg/ml FC: 2.5 μg/ml	Abcam, Cambridge, United Kingdom, Cat# ab181346
Recombinant Rabbit IgG, monoclonal-isotype control	FC: 3 μg/ml	Abcam, Cambridge, United Kingdom, Cat# ab172730
Rabbit monoclonal antibody against UPKIII	IF: 6.75 μg/ml FC: 3 μg/ml	Abcam, Cambridge, United Kingdom, Cat# ab187646

### 2.3 Preparation of the bladder wall, adipose tissue, and buccal oral mucosal tissue

Immediately after collection, tissues were immersed in the DMEM/Ham’s F12 medium (Dulbecco’s modified Eagle medium, HyClone, Logan, UT, United States) supplemented with antibiotics (100 U/ml penicillin, 100 µg/ml streptomycin, and 2.5 µg/ml amphotericin B, Corning, New York, NY, United States; gentamicin 10 μg/ml , Gibco by Life Technologies, Carlsbad, CA, United States). Each tissue fragment was transferred into a separate Petri dish containing DMEM/Ham’s F12 medium. All procedures were performed under sterile conditions.

The urothelium was separated from the underlying stroma and the smooth muscle layer. The collected tissue was cut into pieces (1 cm^2^). The obtained urothelial samples were washed three times in phosphate-buffered saline (PBS, HyClone, Logan, UT, United States) supplemented with antibiotics. Then, they were placed in the DMEM/Ham’s F12 medium.

The adipose tissue was purified by resection of blood vessels. Then, 4 g of adipose tissue was washed three times using PBS supplemented with antibiotics, cut into small pieces, and transferred into a 50 mL centrifuge tube.

In the case of buccal mucosal tissue, the stroma was gently separated from the epithelium. The collected tissue was cut into pieces (2 cm^2^) and washed three times with PBS supplemented with antibiotics. Then, the obtained tissue pieces were placed in the DMEM/Ham’s F12 medium.

### 2.4 Isolation protocols and cell cultures

UCs, BECs, and AD-MSCs were isolated using earlier selected and described methods (Pokrywczynska et al., 2016; Pokrywczynska et al., 2020; Buhl et al., 2021). UCs were isolated using Dispase II (Life Technologies, Carlsbad, CA, United States) according to CELLnTEC manufacturer’s instructions with some modifications (Pokrywczynska et al., 2016; CELLnTEC, 2025a). BECs were isolated using Dispase II and collagenase IV (Life Technologies, Carlsbad, CA, United States) and Accutase (CELLnTEC Advanced Cell Systems, Bern, Switzerland) according to another CELLnTEC manufacturer’s protocol with some modifications (Buhl et al., 2021; CELLnTEC, 2025b). In the case of AD-MSCs, collagenase P was used. The number of isolated cells was determined using the trypan blue exclusion test. UCs and BECs were seeded at a density of 4 × 10^4^ cells/cm^2^ and 2 × 10^4^ cells/cm^2^, respectively. Both types of epithelial cells were cultured in the same commercially available CnT-57 growth medium (CELLnTEC Advanced Cell Systems, Bern, Switzerland) supplemented with 100 U/ml penicillin, 100 µg/ml streptomycin, and 2.5 µg/ml amphotericin B. AD-MSCs were seeded at a density of 2 × 10^4^ cells/cm^2^ and cultured in DMEM/Ham’s F12 medium supplemented with 10% fetal bovine serum (FBS), 10 ng/ml basic fibroblast growth factor (bFGF, Thermo Fisher Scientific, Waltham, MA, United States), 100 U/ml penicillin, 100 $\left(µg\right)/\text{ml}$ streptomycin, and 2.5 µg/ml amphotericin B. Cells were cultured under standard conditions at 37 °C in a 5% CO_2_ atmosphere and 98% humidity. The media were changed every second to third day. UCs, AD-MSCs, and BECs were cultivated in 75 cm^2^ flasks to the third passage. After the second passage, AD-MSCs were additionally seeded in 6- and 12-well culture plates to examine their potential to differentiate. UCs from the third passage were seeded at a density of 4 × 10^4^ cells/cm^2^ in 75 cm^2^ flasks (for continuation of UC-CM collection), 6-well culture plates (for molecular and FC evaluation), and 12-well culture plates (for immunofluorescence examination). AD-MSCs and BECs were seeded at a density of 2 × 10^4^ cells/cm^2^ per well (6-well plates for molecular and FC evaluation; 12-well plates for IF examination). Daily cell morphology and growth were evaluated under an inverted light microscope (Leica, Germany). UCs, BECs, and AD-MSCs cultures with any irregularities (presence of infections, changes in morphology, or cell detachment) were rejected.

### 2.5 Phenotypic analysis

Flow cytometry analyses were performed to characterize the phenotype of UCs and AD-MSCs (FACSCanto II flow cytometer, Becton, Dickinson and Company, Franklin Lakes, NJ, United States). For UC phenotype confirmation, the expression of UC-typical markers (CK7 and UPKIII, Table 2) was analyzed. Phenotypic analysis of AD-MSCs involved cell surface positive markers, including CD90, CD44, and CD29, and negative markers, including CD31, CD11b, and CD45. These markers were localized using the mouse monoclonal antibodies (conjugated to FITC) against CD90 (0.5 μg/ml Exbio, Czech Republic, Praha, Cat# 1F-241-T100), CD44 (0.5 μg/ml , Abcam, Cambridge, United Kingdom, Cat# ab95138), CD29 (0.75 μg/ml , Abcam, Cambridge, United Kingdom, Cat# ab21845), CD31 (0.5 μg/ml , Abcam, Cambridge, United Kingdom, Cat# ab194857), CD11b (0.5 μg/ml , Abcam, Cambridge, United Kingdom, Cat# ab34444), CD45 (0.5 μg/ml , GeneTex, Irvine, CA, United States, Cat# GTX43362), and IgG1 isotype control (0.5 μg/ml , Abcam, Cambridge, United Kingdom, Cat# ab91356). Immunofluorescence (IF) staining was performed to evaluate the BEC phenotype and test whether BECs express CK14 and P63. CK14 expression was confirmed using FC. These markers were localized using a mouse monoclonal antibody against p63 (1:50, Abcam, Cambridge, United Kingdom, Cat# ab735), a goat anti-mouse IgG secondary antibody (1:500, Abcam, Cambridge, United Kingdom, Cat# ab150113), a rabbit monoclonal antibody against CK14 (IF-1:2000, FC-1:190, Abcam, Cambridge, United Kingdom, Cat# ab181595), a goat anti-rabbit IgG secondary antibody (IF-1:500, FC-1:800, Thermo Fisher Scientific, Waltham, MA, Cat# A-11008), and a rabbit IgG isotype control (1:140, Abcam, Cambridge, United Kingdom, Cat# ab199093). The obtained results for all types of cells were developed from three independent cultures.

### 2.6 Adipogenic, osteogenic, and chondrogenic differentiation of AD-MSCs

AD-MSCs were differentiated into adipocytes, osteocytes, and chondrocytes using the Mesenchymal Adipogenesis Kit (Merck Millipore), the StemPro Osteogenesis Differentiation Kit (Life Technologies, Carlsbad, CA, United States), and the StemPro Chondrogenesis Differentiation Kit (Life Technologies, Carlsbad, CA, United States), respectively. Before staining, cells were fixed with 4% formaldehyde (Polysciences, Inc., Washington, PA, United States) for 15 min at room temperature and washed twice with PBS with Ca^2+^ and Mg^2+^ (Corning, New York, NY, United States). After 21-day differentiation into adipocytes, the cells were incubated for 50 min in a 0.37% Oil Red O solution (Sigma-Aldrich, Germany) to stain the lipid vesicles and were rinsed three times with distilled water. Then, the hematoxylin was added for 10 min. After 21-day differentiation into osteocytes, 2% Alizarin red S solution was added for 2 min (pH = 4.2) to stain mineralized nodules. Then, the cells were washed with distilled water. For proteoglycan visualization, AD-MSCs were stained with 1% alcian blue (Sigma-Aldrich) for 30 min and washed with 0.1 N HCl and distilled water. Adipogenic, osteogenic, and chondrogenic differentiation was evaluated under an inverted light microscope (Leica, Germany). The negative control was an AD-MSC culture in a standard medium.

### 2.7 Conditioned medium preparation

The differentiation factor UC-CM was collected from at least 60% subconfluent UC cultures (n = 5) between the first and third passages every day until UCs approached confluency. UC-CM was not filtered. The obtained UC-CM was stored at −80 °C in small aliquots until the mixture was collected from all five pigs. After thawing, it was centrifuged to remove the resulting pellet. For induction of differentiation, UC-CM was mixed with fresh, standard medium (1:1) for AD-MSCs or pBECs.

### 2.8 Differentiation of AD-MSCs and pBECs toward UC-like cells

**FIGURE 1:**
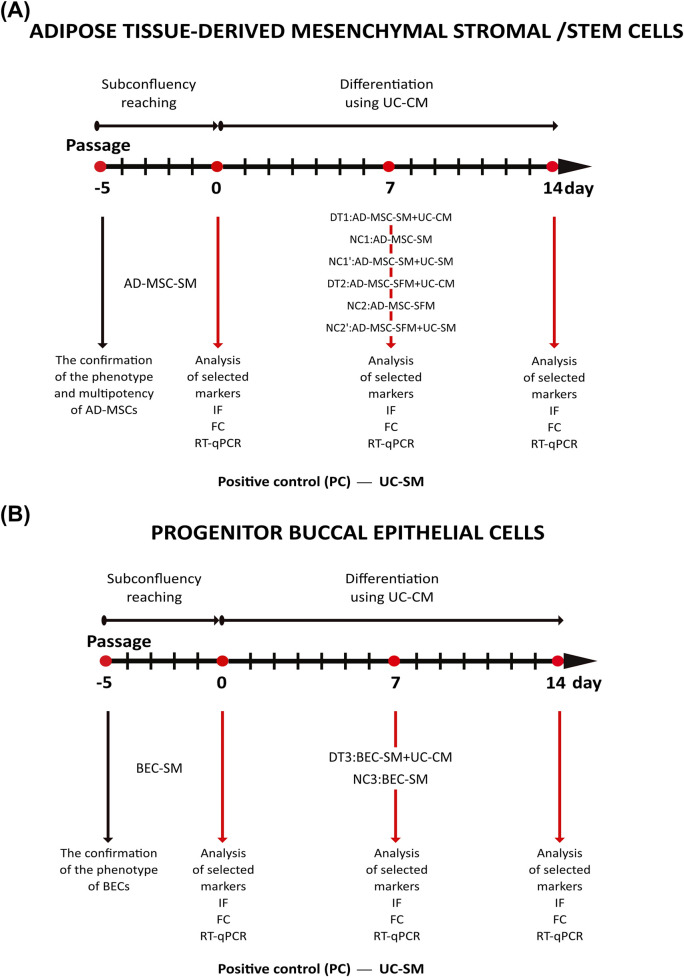
**(A)** AD-MSC and **(B)** pBEC differentiation protocol drafts.

Differentiation of AD-MSCs and pBECs from the third passage was performed by adding the differentiation factor UC-CM (Figure 1). AD-MSCs and pBECs after the third passage were expanded to 80%–90% confluence. From the day they reached subconfluence (“0″ day of the experiment), cultures were exposed to UC-CM for 7 and 14 days to induce differentiation (Figure 1; DT1: AD-MSC-UC-CM and DT3: BEC-SM + UC-CM). Subconfluent UCs derived from the day of the achievement of subconfluence after the third passage were used as a positive control (PC). UC cultures in UC-standard medium (UC-SM) were continued for 7 and 14 days. AD-MSCs and pBECs cultured in their standard media were used as negative controls (NC1: AD-MSC-SM and NC3: BEC-SM). Because the UC culture medium may influence cellular differentiation processes, additional control for AD-MSCs (the mixture (1:1) of their standard culture medium with the unconditioned fresh medium, typically used for UC culture (NC1':AD-MSC-SM + UC-SM, Figure 1A)) was used. The serum could also trigger a differentiation process; therefore, attempts were made to culture AD-MSCs without serum during days 7 and 14 of the experiment (Figure 1A). Then, as a differentiation factor, UC-CM was mixed with the same volume of AD-MSC standard medium without the serum (DT2: AD-MSC-SFM + UC-CM, Figure 1A). Furthermore, AD-MSCs were cultivated as controls in their standard medium without the serum (NC2: AD-SFM) and in the mixture (1:1) of their standard medium without the serum and the unconditioned fresh medium typically used for UC culture (NC2': AD-MSC-SFM + UC-SM, Figure 1A). Daily cell morphology and growth were evaluated under an inverted light microscope (Leica, Germany). Cell morphology for UC and BEC experimental groups (n = 3) was also quantitatively analyzed using cellSens Dimensions 4.2 software (Olympus, Japan). Shading correction and background subtraction were applied to images exhibiting high background levels and uneven illumination to enhance the precision of segmentation. For the FITC channel, an open morphological filter, which combines dilation and erosion operations, was applied to refine object boundaries. Object detection for the DAPI (nucleus) and FITC (cell) channels was executed using a pre-trained neural network pixel classifier integrated into the cellSens software. Objects smaller than 20 μm^2^ or those touching the image border were automatically excluded from the analysis during segmentation. Following this automated process, images were manually reviewed and corrected to address any instances of misdetection or object fusion. Data, including cellular area and elongation, were then extracted and statistically analyzed. For each experimental group, representative examples of segmented objects were selected for visual display. The potential differentiation process progress was assessed on days 0, 7, and 14 (Figure 1).

### 2.9 Gene expression analysis

Cells from all analyzed groups (days 0, 7, and 14) were suspended in PBS without Ca^2+^ and Mg^2+^ (Corning, New York, NY, United States) and stored at −80 °C. The RNA was extracted using the RNeasy Mini Kit according to the manufacturer’s protocol (QIAGEN, Hilden, Germany). cDNA was synthesized using the Transcriptor High Fidelity cDNA Synthesis Kit (Roche Diagnostics, Basel, Switzerland). According to the manufacturer’s instructions, expression of selected genes (cytokeratins: 8, 13, and 19 (*CK8*, *CK13*, and *CK19*), uroplakins (*UPK1A*, UPK1B, UPK3A, and UPK3B), P63, β1 integrin (ITGβ1), cytokeratin 14 (CK14), CD90, and CD105)) was evaluated (n = 5) at the mRNA level by real-time quantitative polymerase chain reaction (RT-qPCR). RT-qPCR was performed using PrimePCR™ primers (Supplementary Table S1, Bio-Rad, Hercules, CA, United States) and the LightCycler 480 SYBR Green I Master Kit (Roche Diagnostics, Basel, Switzerland). The combination of the most stable reference genes (*RPL4* and *TBP*) was selected from β actin (*ACTβ*), hypoxanthine phosphoribosyl transferase 1 (*HPRT1*), ribosomal protein L4 (*RPL4*), ribosomal protein S18 (*RPS18*), and TATA binding protein (*TBP*). Roche LightCycler 480 software V1.5 (Roche Diagnostics, Switzerland) was used to perform advanced relative quantification analysis to evaluate selected marker levels.

### 2.10 Immunofluorescence staining

Immunofluorescent stainings were performed to compare CK7 and UPKIII (Table 2) expression in cells from days 0, 7, and 14 of the experiment (n = 3). For this purpose, cells were fixed by 15 min incubation in a 4% formaldehyde solution (Polysciences, Inc., Washington, PA, United States). After blocking with 4% bovine serum albumin (BSA, Sigma-Aldrich/Merck, Saint Louis, MO, United States) in PBS, cells were stained with primary antibodies (Table 2). Next, secondary antibodies with Alexa Fluor 488 were used (Table 2). The cells were counterstained with DAPI (Sigma-Aldrich/Merck, Germany). The slides were then mounted in Aqua-Poly Mount (Polysciences, United States) and examined using a microscope (Leica, Germany). All acquisitions for selected markers were obtained with the same exposure. Image J-NIH (ImageJ Developers, United States) was used for measuring the fluorescence intensity of individual cells. The resulting data were used to calculate relative cell fluorescence (RCF).

$$RCF=\left(cs\;x\;fic\right)-\left(cs\;x\;fib\right),$$

Where RCF stands for relative cell fluorescence, cs stands for cell surface, fic stands for cell fluorescence intensity, and fib stands for background fluorescence intensity.

### 2.11 Flow cytometry

Cells (1.6 × 10^6^) were fixed in the 4% formaldehyde solution (Polysciences Inc., Warrington, PA, United States) and incubated for 15 min at room temperature. After that, cells were centrifuged and washed twice in PBS. Then, they were permeabilized with 0.5% saponin solution (Sigma-Aldrich, Saint Louis, MO, United States), transferred to −20 °C, and incubated until analysis. On the analysis day, cells were centrifuged and washed in BD Perm/Wash^TM^ (BD Biosciences, Franklin Lakes, NJ, United States). Each suspension was divided into four samples containing 0.4 × 10^6^ cells each, and the samples were washed and centrifuged again. Next, a suitable primary antibody (Table 2) was added to a labeling sample and an isotype control (Table 2) in separate tubes. Cells were incubated at 4 °C in the dark, centrifuged, washed in BD Perm/Wash^TM^ (BD Biosciences, Franklin Lakes, NJ, United States), and suspended with the addition of the secondary antibody (Table 2) (except unlabeled controls). After incubation with the secondary antibody, cells were washed and suspended in BD Perm/Wash^TM^. Stained samples were immediately analyzed using the FACSCanto II Cytometer (BD Biosciences, Franklin Lakes, NJ, United States). A minimum of 5 × 10^3^ events were collected. The obtained results were calculated from four independent samples. The staining index (SI) of the selected marker was analyzed using FACS Diva (BD Biosciences, Franklin Lakes, NJ, United States) and FlowJo^TM^ v10 (BD Biosciences, Franklin Lakes, NJ, United States).

$$\text{SI}=\left(\text{MFI pos}-\text{MFI neg}\right)/\left(2\;x\text{ SDneg}\right),$$

where SI stands for staining index, MFI_pos_ stands for median fluorescence intensity of cells stained using the primary and secondary antibodies, MFI_neg_ stands for median fluorescence intensity of cells stained using the secondary antibody, and SD_neg_ stands for standard deviation for cells stained using the secondary antibody.

### 2.12 Statistical analysis

Data were analyzed using GraphPad Software v8.4 (GraphPad Software, San Diego, CA, United States). Statistical significance was defined as a *p*-value < 0.05. Normality of distribution was analyzed using the Shapiro–Wilk test. Comparisons between groups were made using one-way ANOVA, the Kruskal–Wallis test, and the Tukey and Dunn’s *post hoc* tests.

## 3 Results

### 3.1 Histological and immunohistochemical analysis

HE staining confirmed the presence of urothelium, adipose tissue, and buccal oral epithelium in the examined tissue fragments (Figure 3A). The expression of CK7 was confirmed in all layers of the urothelium within a fragment of the urinary bladder wall. No CK7 expression was found in the adipose tissue or buccal oral mucosa epithelium. UPKIII expression was visible in the suprabasal layers of the urothelium, especially in the superficial layer. The adipose tissue and the buccal oral epithelium exhibited the absence of UPKIII (Figure 3A).

### 3.2 Morphological analysis of established cell cultures

**FIGURE 2:**
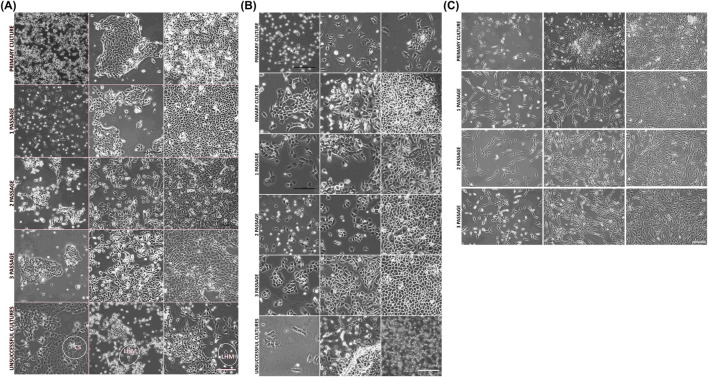
Morphology of UCs, BECs, and AD-MSCs. **(A)** Culture of porcine UCs: primary culture, culture after the first, second, and third passages, and exemplary discarded cultures (unsuccessful cultures) with characteristics of cellular senescence (marked as “CS”) and a lack of homogenous epithelial-typical morphology (marked as “LHM”). **(B)** BEC culture: primary culture, culture after the first, second, and third passages, and exemplary discarded cultures (unsuccessful cultures). **(C)** AD-MSC cultures, including primary culture, culture after the first, second, and third passages. **(A–C)** Inverted microscope, objective magnification ×10, scale bar = 200 µm.

The morphological analysis of cells from established cultures was presented in Figure 2. Unsuccessfully established cultures, including those that were heterogeneous, contaminated with fibroblast cultures, as well as those with infections or other irregularities (such as signs of cell senescence, changes in morphology, poor growth, or cell detachment), were not used in further analyses. Cells from successful cultures maintained the typical morphology until the start of the differentiation trials (Figure 2). UCs and BECs formed a characteristic epithelial “cobblestone”-type pattern (Figure 2A,B), whereas in AD-MSC cultures, a small spindle-shaped typical morphology was observed (Figure 2C).

### 3.3 UC, AD-MSC, and BEC immunophenotypes

**FIGURE 3:**
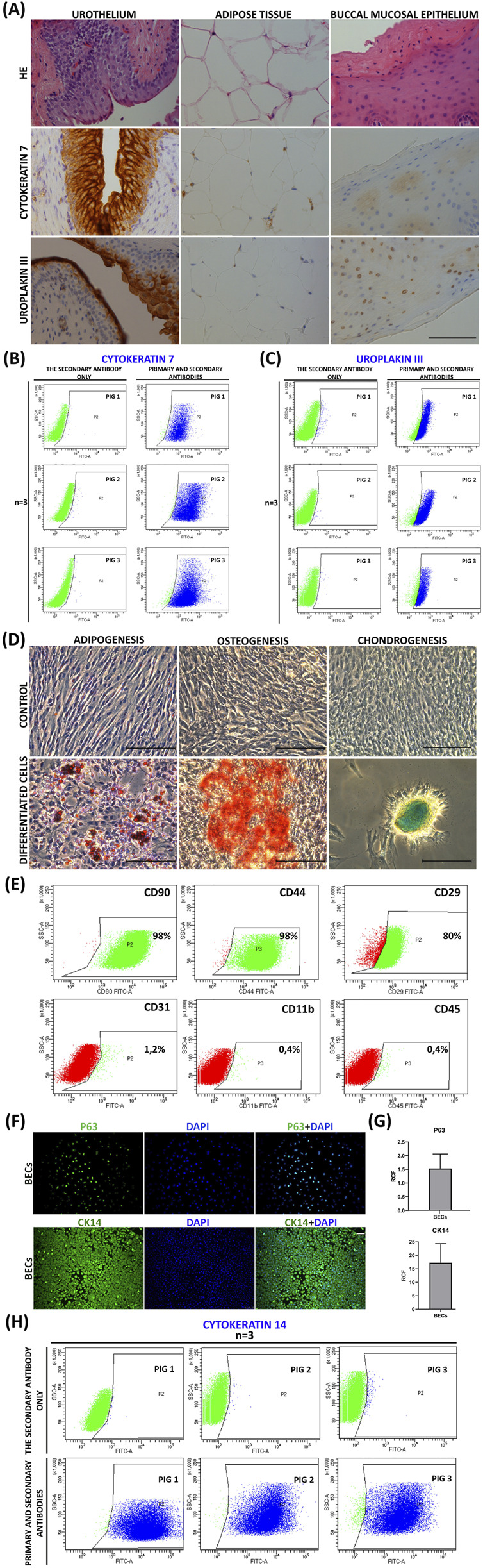
Characterization of the source materials and cell lines that were tested in our study (UCs, AD-MSCs, and BECs). **(A)** Histological and immunohistochemical analysis of a fragment of the bladder wall, adipose tissue, and buccal oral mucosa. Visible expression of CK7 and UPKIII (brown color). Artifactual nuclear changes and artifactual reactions in dying cells are visible in the adipose tissue and buccal oral mucosa. Inverted light microscope, scale = 200 µm. **(B,C)** Analysis of the immunophenotype of UCs (n = 3) using FC. UCs expressing CK7 **(B)**, and UPKIII **(C)** are visible (navy blue). Results obtained using FACS Diva. **(D)** Assessment of adipogenic, osteogenic, and chondrogenic differentiation. Visualized presence of lipid vesicles (oil red O, adipogenesis), mineralized nodules (alizarin red, osteogenesis), and proteoglycans (alcian blue, chondrogenesis). Inverted microscope, bar = 200 µm. FC analysis of the AD-MSC phenotype. Expression of positive markers (green color) and absence of negative expression (red color) were observed. **(E)** FC analysis of the AD-MSC phenotype. Expression of positive markers (green color) and absence of negative expression (red color) were observed. Results obtained using FACS Diva. **(F–H)** Analysis of the immunophenotype of BECs. **(F)** Representative fluorescence P63 and CK14 signals in BECs after the third passage. Fluorescent microscopy; objective magnification ×20 (P63) and ×10 (CK14); scale bar = 100 µm. **(G)** Relative cell fluorescence of P63 and CK14 obtained for BECs. **(H)** Analysis of the immunophenotype of BECs (n = 3) using FC. BECs expressing CK14 are visible (navy blue). Results obtained using FACS Diva.

FC analysis of UCs from the third passage showed positive expression of CK7, which is expressed in all layers of urothelium (Figure 3B) and UPKIII (Figure 3C), which characterizes the superficial cells, in 88.40% ± 9.19% and 95.33% ± 4.10%, respectively (Figure 3B,C). AD-MSCs are characterized by expression of specific surface markers, including CD90, CD44, and CD29, and lack of antigen CD11b, CD31, and CD45 expression. In this study, cultured AD-MSCs from the third passage indicated expression of CD90 (98.23 ± 2.31), CD44 (98.27 ± 1.86), CD29 (80.93 ± 7.01%), CD31 (1.20 ± 0.58), CD11b (0.43 ± 0.25), and CD45 (0.40 ± 0.18%, Figure 3E). BECs from subconfluent cultures after the third passage expressed progenitor BEC-typical markers (P63 protein and cytokeratin 14 (CK14), Figure 3F,G). Furthermore, FC analysis of BECs confirmed positive CK14 expression (Figure 3H). CK14 is the marker of all oral epithelial tissue cell layers from buccal mucosa.

### 3.4 Assessment of AD-MSC differentiation potential

The formation of lipid vesicles (oil red O, adipogenesis), mineralized nodules (alizarin red, osteogenesis), and proteoglycans (alcian blue, chondrogenesis) was observed, which confirmed, respectively, the adipogenic, osteogenic, and chondrogenic potential of cultured AD-MSCs (Figure 3D). Control AD-MSCs cultivated in a standard medium did not indicate differentiation features (Figure 3D).

### 3.5 Assessment of the differentiation protocol through morphological analysis

**FIGURE 4:**
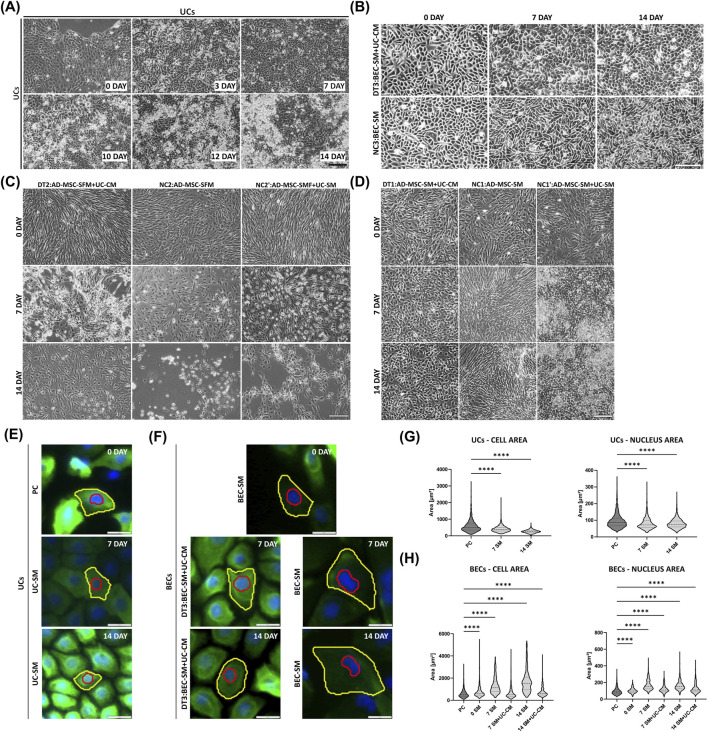
Morphology of UCs, BECs, and AD-MSCs during the 14-day experiment. **(A)** UCs in the SM during the 14-day experiment. **(B)** BECs cultured in a mixture of SM and CM from UC cultures (DT3) and in SM (NC3) on day 0 and after differentiation periods (on days 7 and 14 of the experiment). **(C)** AD-MSCs cultured in a mixture of SM without the serum and UC-CM (DT2), in SM without serum (NC2), and a mixture of SM without the serum and SM for UCs (NC2’) on day 0 and after 7 and 14 days of differentiation. **(D)** AD-MSCs cultured in a mixture of SM and UC-CM (DT1), in SM (NC1), and a mixture of SM and the SM for UCs (NC1’) on day 0 and after 7 and 14 days of differentiation. **(A,B)** Typical epithelial cell morphology with characteristic “cobblestone”-type pattern. **(C,D)** Fibroblast-like spindle-shaped cells on day 0. **(A,D)** Visible local stratification from the day 10 for UCs and after 7 and 14 days of exposure to the unconditioned CnT-57 medium for AD-MSCs (NC1’). **(D)** Change from a fibroblast-like morphology to an epithelial-like morphology in AD-MSC cultures in the mixture of SM and conditioned/unconditioned CnT-57 (DT1 and NC1′) after 7 and 14 days of differentiation. **(C)** Detachment of cells after 7 and 14 days of the experiment. Inverted microscope, scale bar = 200 µm. **(E,F)** Examples of cells representing the average parameters of the analyzed cell population on day 0 and after 7 and 14 days in the experimental groups: PC; UCs cultivated in the SM; and BECs cultivated in the SM and DT3. Scale bar = 200 µm. **(G,H)** Truncated violin plots performing the quantitative analysis of cell morphology (cell area and cell nucleus respectively) for 0-, 7-, and 14-day cultures of **(G)** UCs (PC and UCs cultivated in the SM) and **(H)** BECs (cultured in the SM and SM + UC-CM). Multiple comparisons between PC and the other experimental groups. ****p < 0.0001.

The morphology of cells observed during the experiment is presented in Figure 4. Both control and tested epithelial cells (UCs and pBECs) maintained their typical epithelial morphology until the end of the experiment (Figure 4A,B). In UCs, the local stratification was observed from the 10th day (Figure 4A). AD-MSCs cultured in a mixture of standard medium and conditioned/unconditioned CnT-57 medium showed a change from fibroblast-like morphology to an epithelial-like morphology after 7 and 14 days of differentiation (DT1 and NC1’ Figure 4D). The difference was observed as the local stratification in AD-MSC cultures after 7 and 14 days of exposure to the unconditioned CnT-57 medium (NC1’ Figure 4D). In contrast, when cultivated using their standard medium, AD-MSCs maintained the characteristic spindle shape in 7- and 14-day cultures (NC1, Figure 4D). In the case of AD-MSCs cultured in media without serum, cell detachment was observed (DT2, NC2, and NC2’, Figure 4C). Only a portion of the cells remained attached to the culture surfaces on the last day of the experiment (day 14, NC2 and NC2’, Figure 4C). BEC cultures in the medium containing UC-CM (DT3) and in their standard medium (NC3) formed characteristic “cobblestone”-type patterns throughout the experiment. They did not detach from the surface of the culture vessels (Figure 4B). The quantitative analysis of cell morphology (cell area and cell nucleus, respectively; Figures 4E–G) for 0-, 7-, and 14-day UC and BEC cultures indicated that UCs from the PC had larger surface and nucleus areas than UCs from 7- (*p* < 0,0001) and 14- (*p* < 0,0001) day cultures (Figures 4E–G). BECs from day 0 of the experiment had a significantly larger surface area than PC, in contrast to pBECs from day 7 of the experiment, which were differentiated using a conditioned medium (7 SM + UC-CM) that achieved a similar surface area to the PC (*p* > 0.05; Figure 4H). However, pBECs from day 14 of the experiment, also exposed to the differentiation factor (14 SM + UC-CM), had a significantly larger surface area than the PC (*p* < 0.0001, Figure 4H), similar to other BEC-analyzed groups. The nucleus had a substantially larger surface area in BEC from every analyzed group than in the PC (*p* < 0.0001, Figure 4H).

### 3.6 Assessment of the differentiation protocol through molecular analysis of selected urothelial marker expression

**FIGURE 5:**
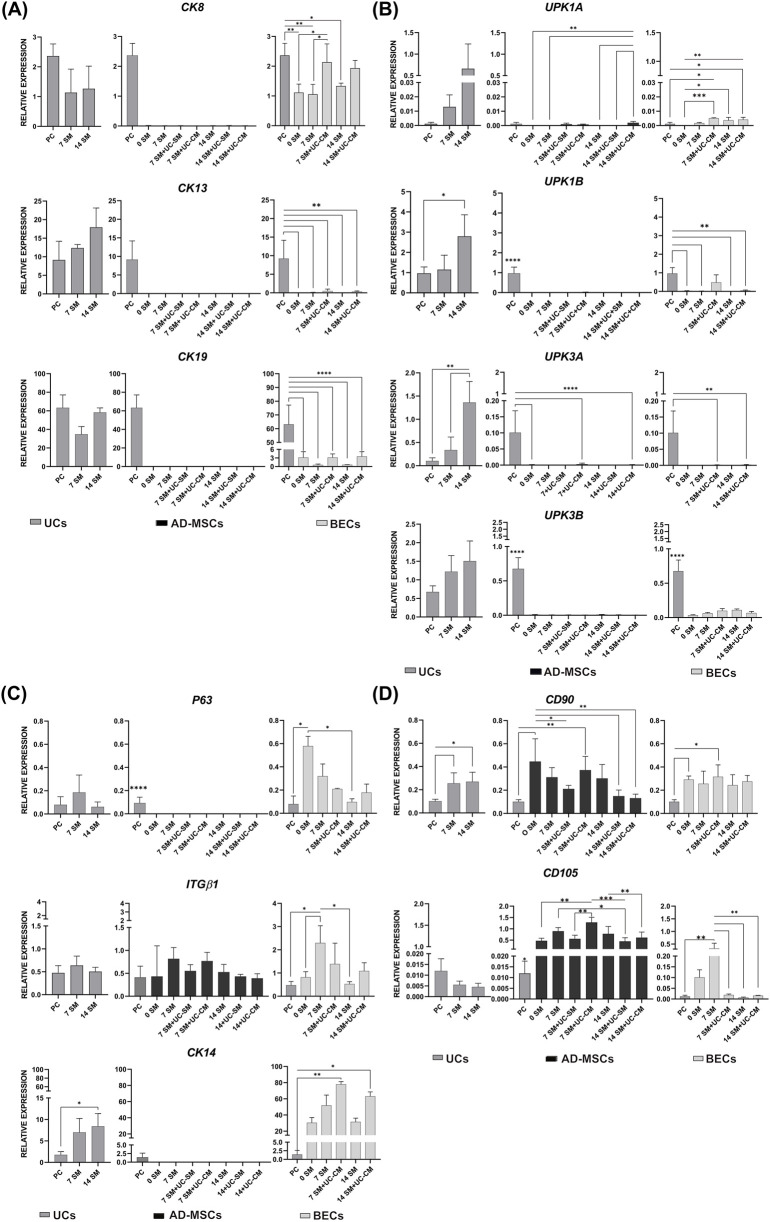
Expression of **(A,B)** urothelial (*CK8*, *CK13*, *CK19*, *UPK1A*, *UPK1B*, *UPK3A*, and *UPK3B*), **(C)** progenitor epithelial (*P63* and *ITGβ1*), *CK14*, and **(D)** mesenchymal cell markers (*CD90* and *CD105*) in UCs, AD-MSCs, and BECs exposed to different media conditions (SM/SM + UC-CM) from day 0 of the experiment for 7 and 14 days. *CK8*, *CK13*, and *CK19* results in BECs are presented as means and standard deviations. *CK19* results in UCs are presented as medians and ranges. *UPK1A* and *UPK1B* results in UCs are presented as medians and ranges. *UPK1A* and *UPK1B* results in AD-MSCs are presented as means and standard deviations. All *UPK3A* and *UPK3B* results are presented as means and standard deviations. *P63* results in UCs and BECs are presented as medians and ranges, and *ITGβ1* results are presented as means and standard deviations. *ITGβ1* results in AD-MSCs are presented as medians and ranges. *CK14* results in UCs are presented as means and standard deviations, and those in BECs as medians and ranges. All *CD90* and *CD105* results are presented using means and standard deviations. Multiple comparisons between all UC, AD-MSC, and BEC experimental groups and PC were performed. Analyses by RT-qPCR. *p < 0.05. **p < 0.01. ***p < 0.001.****p < 0.0001.

Markers from the cytokeratin group are crucial proteins characterizing epithelial cells. RT-PCR analysis revealed expression of the urothelial umbrella cell *marker CK8* and basal layer UC markers *CK13* and *CK19*, in UCs from day 0, 7, and 14 cultures. In contrast, these urothelial marker expressions were absent in all tested AD-MSC cultures (Figure 5A). In the case of BECs cultured in their standard medium, *CK8* expression was on the same level after 0, 7, and 14 days and was significantly lower than in the PC (*p* < 0.05, PC). BECs cultured using a conditioned medium indicated comparable *CK8* expression to PC (Figure 5A). In the case of BEC cultures, a lack or minimal expression of *CK13* was observed; only in the group in which pBECs were differentiated using conditioned medium were higher levels of *CK13* expression observed (*p* > 0.05, Figure 5A). These results indicate that adding the differentiation factor increased *CK8* and *CK13* expression in BECs. Simultaneously, it suggests an increased differentiation stage toward urothelial-like cells for BECs subjected to the urothelial differentiation protocol. On the contrary, the influence of the differentiation factor on AD-MSCs was insufficient to induce the expression of the indicated urothelial markers (Figure 5A). *CK19* expression in BECs from day 0 was low but noticeable. There were no significant differences in *CK19* expression levels among the BEC groups analyzed in this experiment (Figure 5A), including BECs cultured with the addition of UC-CM.

The expression of specialized UC markers (*UPK1A*, *UPK1B*, *UPK3A*, and *UPK3B*) was observed in UCs from all experimental cultures. UCs from day 14 of the experiment achieved the highest expression levels for all analyzed uroplakins, confirming the highest differentiation level of these cells. However, statistical analysis confirmed significant differences only for *UPK1B* (*p* < 0.05) and *UPK3A* (*p* < 0.01, Figure 5B). AD-MSCs from day 0 had no or barely-detectable uroplakin levels. The UC-CM addition increased *UPK1A* in AD-MSCs from day 14 compared to day “0” (*p* < 0.01, Figure 5B). BECs from day “0” expressed no or barely-detectable uroplakin levels (Figure 5B). *UPK1A* expression significantly increased in 7- and 14-day BEC cultures (*p* < 0.05), except for 7-day cultivation using the standard medium (*p* > 0.05, Figure 5B). In BECs cultured in conditioned medium environment derived from day 7, *UPK1B* expression reached a level comparable to that in UCs from day “0” (*p* > 0.05, PC), which may indicate an ongoing differentiation process toward urothelial-like cells. *UPK1B* expression was significantly lower in the tested BEC groups than in the PC (*p* < 0.01, Figure 5B). There were no differences between the expression levels of either *UPK3A* or *UPK3B* in all groups of BECs (*p* > 0.05, Figure 5B). It should be observed that RT-PCR analyses showed expression (including its barely-detectable levels) of the uroplakin genes *1A*, *1B*, and *3B* in more tested BEC cultures than in AD-MSC cultures (Figure 5B). Such observations could be essential in comparing the differentiation potential of pBECs and AD-MSCs toward urothelial-like cells.

### 3.7 Assessment of the differentiation protocol through molecular analysis of selected progenitor epithelial cell marker expression

BECs expressed selected progenitor cell markers (*P63*, *ITGβ1*, and *CK14*) in all examined cultures (Figure 5C). *P63* expression decreased in BECs from 7- and 14-day cultures compared to cultures from day 0 of the experiment; however, a statistically significant decrease was observed only in BECs cultured in the standard medium between days 0 and 14 of the experiment. The results of *P63* expression in BECs suggest a higher number of cells with more differentiated stages in BEC cultures from days 7 and 14 compared with day 0 of the experiment. Additionally, the analyses showed a statistically significantly higher *P63* expression in BECs from day 0 compared to PC (Figure 5C). Similarly to *P63* expression, it was assumed that the level of *ITGβ1* expression should decrease as a result of the differentiation of pBECs. However, *ITGβ1* expression was maintained at a comparable level in most of the tested BEC cultures. There was also no significant difference between the expression values of this gene between BECs and PC (*p* > 0.05, Figure 5C). The expression of another progenitor cell marker, *CK14*, was much higher in BECs from all examined groups than in PC. In the case of BECs cultured in the standard medium, it was just a visible trend without confirmation by statistical analysis (p > 0.05, Figure 5C). On the contrary, a significant difference was observed between PC and BECs cultured using a conditioned medium. Upon interaction with UC-CM, *CK14* expression in BECs increased (*p* < 0.05, Figure 5C). AD-MSCs did not express *P63* or *CK14* in any of the tested groups. The addition of the differentiation factor did not affect the appearance of these markers (Figure 5C). In contrast, *ITGβ1* expression was found in AD-MSCs in all examined cultures (Figure 5C).

### 3.8 Assessment of the differentiation protocol through molecular analysis of selected mesenchymal cell marker expression

Expression of selected mesenchymal cell markers (*CD90* and *CD105*) was confirmed in AD-MSCs from all examined groups (Figure 5D). The highest *CD90* expression was observed in AD-MSCs from day 0. On day 14 of the experiment, the expression of this marker decreased (Figure 5D), which may indicate the success of the differentiation protocol. The highest *CD105* expression was observed after 7 days of AD-MSC cultivation in the UC-CM environment. This result differs the most from the other (lower) results obtained for the tested AD-MSC cultures. On day 14 of the experiment, the expression of *CD105* in the group with the addition of conditioned medium returned to the beginning level (Figure 5D). UCs from day 0 expressed *CD90*, which was upregulated after 7 and 14 days of culture (*p* < 0.05, Figure 5D). *CD105* expression had barely detectable levels in all examined UCs (Figure 5D). The expression of *CD90* and *CD105* was present in the tested BECs (Figure 5D).

### 3.9 Assessment of the differentiation protocol through immunofluorescence analysis of selected urothelial marker expression

**FIGURE 6:**
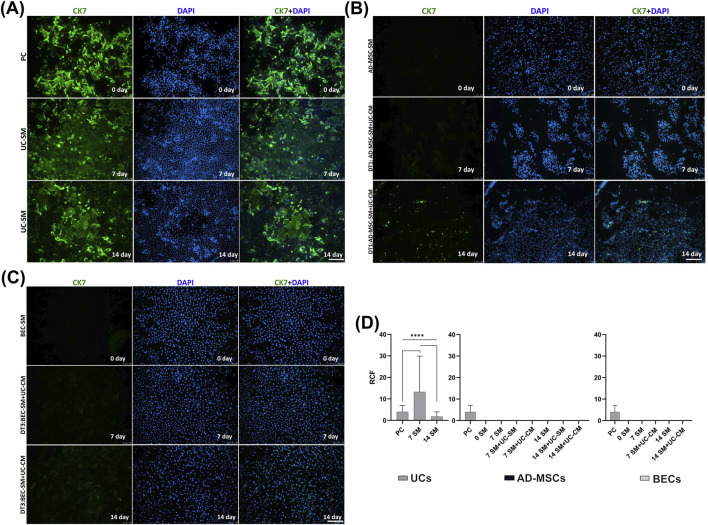
Representative fluorescence signals of CK7 and DAPI in **(A)** UC experimental groups: PC; UC-SM—cells with positive CK7 expression are visible (green)), **(B)** AD-MSC experimental groups: AD-MSC-SM; DT1— artifactual reactions of dying cells are visible, and **(C)** BEC experimental groups: BEC-SM; DT3 on days 0, 7, and 14 of the experiment. Fluorescent microscopy, scale bar = 200 µm. **(D)** RCF after labeling with the antibody against CK7 of 0-, 7-, and 14-day UC, AD-MSC, and BEC cultures (SM/SM + UC-SM/SM + UC-CM). Analyses were performed as multiple comparisons between all UC, AD-MSC, and BEC experimental groups and PC as median and range. ****p < 0.0001.

UCs from all analyzed groups showed positive expression of CK7 (Figures 6A,D), which is the early marker of UCs. The highest RCF was observed in UCs derived after day 7 of the experiment (*p* < 0.0001, Figures 6A,D). The presence of CK7 was not confirmed in any of the analyzed AD-MSC and BEC cultures (Figures 6B–D, 8), including cells subjected to urothelial differentiation.

**FIGURE 7:**
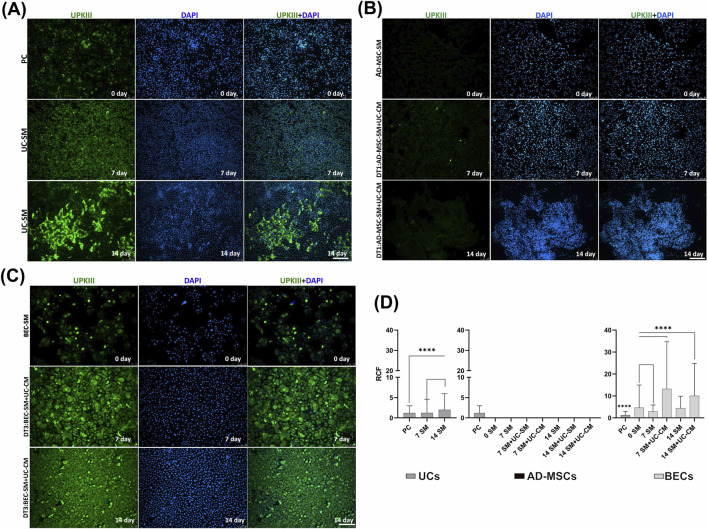
Representative fluorescence signals of UPKIII and DAPI in **(A)** UC experimental groups: PC; UCs in the SM (UC-SM), **(B)** AD-MSC experimental groups: AD-MSC culture in the SM (AD-MSC-SM; DT1)—artifactual reactions of dying cells are visible, and **(C)** BEC experimental groups: BEC-SM; DT3 on days 0, 7, and 14 days of the experiment. **(A)** UCs and **(C)** BECs with positive UPKIII expression are visible (green). Fluorescent microscopy, scale bar = 200 µm. **(D)** RCF after labeling with the antibody against UPKIII of 0-, 7-, and 14-day UC, AD-MSC, and BEC cultures (SM/SM + UC-SM/SM + UC-CM). Analyses were performed as multiple comparisons between all UC, AD-MSC, and BEC experimental groups and the PC as median and range. ****p < 0.0001.

In all tested groups, UCs exhibited the expression of UPKIII (Figures 7A,D), a marker which is part of the specialized UC phenotype. UCs from day 14 of the experiment expressed significantly higher levels of UPKIII than UCs from days 0 (PC) and 7 (*p* < 0.0001, Figures 7A,D). In AD-MSCs, a lack of UPKIII was noticed (Figures 7B,D,9A,B). Expression of UPKIII was observed in BECs (Figures 7B,D,9C). After both differentiation periods (7 and 14 days), the level of UPKIII expression increased in BECs subjected to urothelial differentiation (*p* < 0.0001, Figures 7B,D). This effect was not visible in the case of BECs cultured in a standard medium after days 7 and 14 of the experiment (Figures 7D,9C). Interestingly, even UPKIII expression in BECs cultured in the standard medium was higher than in PC (*p* < 0.0001, Figures 7A,D,9C).

### 3.10 Assessment of the differentiation protocol through flow cytometry analysis of selected urothelial marker expression

UCs from days 0, 7, and 14 showed the presence of the early marker of UCs, CK7, at a similar level (*p* > 0.05, Figures 10A,B). AD-MSCs did not indicate CK7 expression (Figures 10A,C). CK7 expression was demonstrated in BECs from the tested cultures but with significantly lower values than in the PC (*p* < 0.05, Figures 10A,D). No statistically significant increase in the SI value between BECs cultured in a standard medium and with the addition of UC-CM was observed (*p* > 0.05, Figure 10A), which shows that the attempt to induce early UC marker expression in the presented study was unsuccessful.

The expression of urothelial-specific cell marker, UPKIII, was confirmed in UCs (Figures 10E,F). No significant differences were found between the SI values for UPKIII in UCs from days 0, 7, and 14 of the experiment (*p* > 0.05, Figure 10E). The results of the UPKIII level obtained for UCs and AD-MSCs from day 0 were comparable (*p* > 0.05, Figures 10E–G). The results from AD-MSCs from all cultures derived from days 7 and 14 were significantly lower than for AD-MSCs from day 0 (*p* < 0.01, Figure 10E). The use of the differentiation factor in AD-MSCs did not trigger an increase in the UPKIII level (*p* < 0.01, Figures 10E,G). Performed analyses revealed UPKIII expression in BECs (Figures 10E,H). SI values received for BECs from day 0 and from cultures subjected to urothelial differentiation were lower than for BEC cultures cultured in the standard medium (*p* < 0.05, Figures 10E,H). It suggests that the unconditioned CnT-57 medium affects the differentiation of BECs toward UCs. The results that are more similar to the norm (PC) were obtained for BECs cultivating using UC-CM (Figures 10E,F,H).

## 4 Discussion

In many diseases requiring urinary tract reconstruction (invasive bladder cancer and interstitial cystitis), it is difficult or impossible to obtain autologous UCs (Atala et al., 2006; Southgate et al., 2007). In reconstructive urology, easily available undifferentiated cells (AD-MSCs/BM-MSCs), which trigger signaling pathways to regenerative processes, are suitable only for application in urinary tract augmentation (the urinary tract wall reconstruction). The construction of a neo-bladder or neo-conduit using tissue engineering techniques requires a high density of differentiated urinary bladder smooth muscle and urothelial cells or stem cells (Pokrywczynska et al., 2018; Pokrywczynska et al., 2017; Pokrywczynska et al., 2019; Pokrywczyńska et al., 2018; Buhl et al., 2019; Adamowicz et al., 2021).

In our previous work, we used the Bladder Acellular Matrix (BAM) grafts seeded with AD-MSCs for the regeneration of the porcine urinary bladder wall. The analysis of the expression of UC and SMC markers by RT-qPCR did not confirm the complete regeneration of the reconstructed fragments. Moreover, the cytotoxic effect of urine led to cell death (Pokrywczynska et al., 2018). UCs are less sensitive to urine than AD-MSCs, which is why UC-seeded scaffolds could protect against urine penetration into deeper layers of the graft and prevent damage to cells seeded simultaneously on the outer scaffold side (Pokrywczyńska et al., 2018). Therefore, there is a need to find an alternative UC source for the construction of urinary tracts *de novo*.

In this report, two potential cell sources for tissue engineering in urology were tested: porcine subcutaneous adipose tissue of the abdominal wall and buccal mucosal tissues, from which AD-MSC and BEC cultures were established, respectively. As we mentioned earlier, adipose tissue is an extremely attractive material for isolating potential cells for urinary tract reconstruction. AD-MSCs are characterized by their easy availability, confirmed capacity for multidirectional differentiation, and a distinct panel of markers for identification (Viswanathan et al., 2019; Gronthos et al., 2001; Linard et al., 2018). Epithelial cells exhibiting stemness characteristics are located in the oral mucosa (Wang et al., 2019; Hustler et al., 2018; Calenic et al., 2015; Calenic et al., 2014; Buhl et al., 2021; Boldrup et al., 2011). To date, the most common attempts have been to establish and characterize epithelial progenitor cell cultures derived from the gingival mucosa (Jones and Klein, 2013; Calenic et al., 2010). However, in the present study, pBECs were tested for differentiation into UCs, both due to the easy accessibility of buccal mucosa and the ease of obtaining a small tissue biopsy for BEC isolation. The potential of buccal mucosa is already being utilized in urology for the treatment of urethral strictures (Hillary et al., 2014; Ławkowska et al., 2023; Smith, 2016). To date, no guidelines have been established for confirming the phenotype of these cells, which makes their identification difficult (Jones and Klein, 2013; Hustler et al., 2018). However, previous studies conducted by our team have provided promising results in the isolation, culture, and confirmation of the phenotype of BECs. Porcine BECs have been shown to express epithelial stem/progenitor cell markers, including CK14, P63, and ITGβ1, along with the SHH protein (Buhl et al., 2021). SHH protein expression is significant in the context of using BECs in urinary tract reconstruction. SHH is a signaling protein that plays a key role in bladder organogenesis, including interactions between the developing, mutually stimulating layers of urothelium and muscularis (Baskin et al., 1996). In this report, both cell lines, including AD-MSCs and BECs, were successfully isolated. AD-MSCs exhibited the typical immunophenotype characteristic of porcine mesenchymal stromal/stem cells. The expression of progenitor cell markers characterized BEC cultures (Figures 3E–H). These cell cultures, established and subsequently subjected to differentiation trials, were heterogeneous in nature. They were co-cultures of progenitor and differentiated cells. The development of an effective *in vitro* cell transdifferentiation protocol remains unavailable (Ahrens et al., 2019). Therefore, we assumed that the urothelial-like phenotype, potentially resulting from the experiment, would result from the differentiation of pBECs, despite the possibility of transdifferentiation.

AD-MSC and BEC cultures were exposed to UC-CM to induce differentiation. In the future, identifying the components of conditioned media will allow for the design of a chemically defined medium that induces other phenotypes. However, identifying the components of the UC-CM was beyond the aim of this study. Additionally, due to a lack of reagents for testing cells from domestic pigs, it is currently impossible to perform such an analysis (Turner, 2015; Alvarez-Dominguez and Melton, 2022; Descamps and Emanueli, 2012; Raman et al., 2022; Drewa et al., 2013). Other frequently used methods of stem cell differentiation include culture using a chemically defined medium, co-culture, and mechanotransduction. The co-culture and CM use are based on delivering factors from adult cells to stem/progenitor cells. CM collection is time-consuming, but it has real potential for clinical use because of the simple storage method and the straightforward addition of the medium in the appropriate proportion to the stem cell culture (Turner, 2015; Alvarez-Dominguez and Melton, 2022; Descamps and Emanueli, 2012; Raman et al., 2022; Drewa et al., 2013).

Data on earlier attempts to differentiate AD-MSCs/BM-MSCs toward urothelial-like cells using UC-CM have been reported, but their findings were inconsistent (Liu et al., 2009; Shi et al., 2012; Al.-kurdi et al., 2021; Tian et al., 2010; Zhang et al., 2014). MSCs showed increased urothelial marker (CK7, CK13, CK19, UPK1a, and UPK2) expression levels due to UC-CM addition (Shi et al., 2012; Al.-kurdi et al., 2021; Tian et al., 2010; Zhang et al., 2014). In contrast, some results show that this differentiation factor is insufficient to initiate the differentiation of AD-MSCs. Liu et al. (2009) demonstrated that after the exposition of AD-MSCs to the UC-CM environment, CK18, UPK1B, and UPK2 expression was not observed.

In this study, AD-MSC and pBEC cultures were exposed to UC-CM mixed with standard medium in a 1:1 ratio for 7 and 14 days to induce differentiation. Zhang et al. (2014) used a comparable approach, collecting UC-CM every 24 h. They cultured AD-MSCs for 21 days in the mixture (1:1) of their standard culture medium and the CM (Zhang et al., 2014). Shi et al. (2012) and Tian et al. (2010) differentiated AD-MSCs and BM-MSCs for 14 days using a mixture of UC-CM (collected previously every 12 h) and standard medium in a ratio of 3:1. In Al.-kurdi et al. (2021), the differentiating factor (for 2 weeks) was the UC-CM added in a 1:4 ratio to DMEM containing 10% FBS or 2.5/5% human platelet lysate (HPL).

In this report, AD-MSCs changed from a fibroblast-like morphology to a epithelial-like shape due to differentiation using UC-CM with the addition of fresh CnT-57 (Figure 4D). In contrast, other studies reported that only 25%–50% of AD-MSCs adopted a shape typical of epithelial cells after exposure to the differentiating factor (Shi et al., 2012). In our report, confluence was not the cause of the disappearance of the fibroblast-like morphology. AD-MSCs cells maintained their fibroblast-like shape in cultures grown in a standard medium (NC1, Figure 4D). During the 14 days of the experiment, most AD-MSCs cultivated in serum-free cultures lost their fibroblast-like shape and detached (AD-MSC-SFM, Figure 4C). These cultures were not further analyzed. The serum was necessary for the growth of AD-MSCs. Comparable findings were recently reported by Al.-kurdi et al. (2021), who presented similar observations. In their study, the differentiating factor for AD-MSCs was UC-CM combined with DMEM supplemented with 10% FBS or 2.5/5% pooled human platelet lysate (pHPL). The highest expression levels of UC-typical markers were observed in AD-MSC cultures in medium with added FBS. Meanwhile, using UC-CM supplemented with pHPL resulted in low or absent expression of the analyzed UC-typical markers (Al.-kurdi et al., 2021).

To date, no attempt has been made to generate urothelial-like cells from pBECs. Therefore, we cannot compare our results of the morphology assessment (Figure 4B) or any other results of BECs cultured under differentiation conditions.

IF stainings indicated that the early marker of urothelial cells, CK7, was present only in the established UC cultures (Figure 6). In contrast to IF results, FC analysis showed expression of CK7 also in BECs from day 0 (Figures 10A,D). Both techniques demonstrated that attempts to induce increases in CK7 by exposing pBECs to the UC-CM environment were unsuccessful (Figures 6, 10A,D). The results of Hustler et al. (2018) showed that human BECs cultured in a medium with similar characteristics to the CnT-57 medium used in this study (no serum, low concentration of calcium ions) expressed CK7. CK7 expression was also not detected after exposition of AD-MSCs to the differentiation factor (Figures 10A,C). In contrast to the results of the current study, Tian et al. (2010) obtained increased CK7 expression in BM-MSCs that were differentiated toward UC cells during the 14-day experiment using the UC-CM.

In the present study, BECs exhibited the presence of the urothelial umbrella cell marker, *CK8*, similar to that in PC (*p* > 0.05, Figure 5A) after 7 and 14 days of exposure to the differentiation factor. Low-level expression of CK13, located in the urothelium’s basal layer, was observed in BECs treated with UC-CM (Figure 5A). Our examination did not reveal the expression of *CK8*, *CK13*, and *CK19* in AD-MSCs (Figure 5A). In Tian et al. (2010), contradictory results were obtained—an increase in CK13 expression was demonstrated in BM-MSCs after a 14-day exposure to the UC-CM. Al.-kurdi et al. (2021) recently presented a more than four-fold increase in CK19 expression in AD-MSCs after culture in the UC-CM environment.

In analyzed cells, absent or low expression of *UPK1a* was observed (Figure 5B). Other authors presented outcomes that did not align with our findings, designing the protocols that enable the detection of *UPK1A* expression in UCs. Their results also indicated the increase in this marker in BM-MSCs and AD-MSCs undergoing differentiation toward UCs (Tian et al., 2010; Zhang et al., 2014). Uroplakin *1b* can be detected in UCs with a lower degree of differentiation than umbrella cells and in corneal and airway epithelial cells (Hustler et al., 2018; Adachi et al., 2000; Olsburgh et al., 2003). In the presented study, the expression of *UPK1b* was confirmed for BECs after 7 days of exposure to the differentiation factor (Figure 5B). The results obtained for AD-MSCs were consistent with those reported by Liu et al. (2009), in which the expression of *UPK1B* was not observed after differentiation with CM (Liu et al., 2009). *UPK3b*, similar to *UPK1b,* is located in less differentiated UCs (Hustler et al., 2018). Moreover, it is expressed by different epithelial cell types (not only UCs) (Kanamori-Katayama et al., 2012; Rudat et al., 2014), which was confirmed in this study—*UPK3b* was expressed by BECs (Figure 5B).

Immunofluorescence analyses showed the highest UPKIII expression in BECs from day 7 of exposure to the differentiation factor (Figures 7C,D). Expression of this marker was not observed in AD-MSCs (Figures 7B,D,9A,B). Recently, Liu et al. (2009) presented similar results. After exposure to the differentiation factor, AD-MSCs showed no expression of UPKIII (Liu et al., 2009). In the present study, UPKIII was unexpectedly detected in AD-MSCs using flow cytometry; the expression levels of UPKIII in day 0 AD-MSCs and the positive control were similar (*p* > 0.05, Figures 10E,G). This result is unclear and requires additional studies. UPKIII expression in BECs cultured for 7 and 14 days in a standard medium was significantly higher than for PC (*p* < 0.05, Figures 10E,H). It shows that unconditioned CnT-57 medium affects the increase in differentiation of BECs toward UCs. For epithelial cultures, we used the commercially available Cnt-57 medium—its efficiency was confirmed earlier (Pokrywczynska et al., 2016; Buhl et al., 2021; Buhl et al., 2019; Adamowicz et al., 2021). The CnT-57 medium, despite the lack of serum, is not chemically defined due to the supplementation with bovine pituitary extract (BPE). In addition, the mixture of other ingredients in this medium (the company’s confidential data) was composed for the maximum attachment and proliferation of epithelial cells. Therefore, using the commercially available CnT-57 medium can be associated with the possibility of affecting the phenotype of target cells. AD-MSCs were cultured in the DMEM/Ham’s F12 medium supplemented with 10% FBS, bFGF, and antibiotics. Our earlier results confirmed the optimal growth of cells and repeatable results obtained with this medium (Pokrywczynska et al., 2020). However, transferring this method to clinical practice would require changing the medium to serum-free.

We assumed that the expression level of *P63* in BECs would decrease during differentiation. However, only the decrease between BECs cultured in BEC-SM from days 0 and 14 was statistically significant (*p* < 0.05). The nature of stem/progenitor cell character is also reflected in the expression of *ITGβ1* (Calenic et al., 2015; Southgate et al., 1999; Campbell and Humphries, 2011; Schnittert et al., 2018; Southgate et al., 1987; Jones and Watt, 1993). The obtained results indicated that expression of this marker in BECs cultured using UC-CM was maintained at the beginning level (*p* > 0.05, Figure 5C). Interestingly, UCs and AD-MSCs from day 0 were characterized by similar *ITGβ1* expression values (*p* > 0.05, Figure 5C). The presence of integrins in mesenchymal cells was previously demonstrated by Kräter et al. (2017).

We also hypothesized that it would be possible to distinguish BECs from UCs by the presence or lack of *CK14*. In this study, the expression of *CK14* was found both in BECs and UCs (Figure 5C). Our experiment was performed on porcine cells, while other authors analyzed the potential presence of *CK14* in the human UCs and BECs (Wang et al., 2019; Hustler et al., 2018). Other explanations for *CK14* expression in UC and BEC cultures may be the low concentration of calcium ions and the lack of serum in the CnT-57 medium, which was the standard medium to culture both UCs and BECs in this study. Hustler et al. (2018) observed that UCs cultured in a medium with such characteristics express cytokeratin 14 and have lost the expression of most of the markers typical for specialized UCs (Hustler et al., 2018; Fishwick et al., 2017; Varley et al., 2005).

The evaluation of the differentiation process also included the analysis of essential markers of mesenchymal cells—*CD90* and *CD105* (Viswanathan et al., 2019; Yoon and Ho, 2019). The *CD90* and *CD105* expressions were expected to decrease during AD-MSC differentiation toward UC cells. The observed decrease in CD90 expression on day 14 of differentiation supported this expectation. *CD90* expression was additionally found in all examined UC and BEC cultures (Figure 4D), which aligns with the findings of Nakamura et al. (2006), who identified *CD90* expression in keratinocytes. The role of *CD105* in mesenchymal cells has yet to be determined (Schoonderwoerd et al., 2020). UC-CM did not induce a decrease in the expression of this marker in AD-MSCs (Figure 5D). Our results indicated minimal *CD105* expression in the positive control and low expression in BECs from days 0 and 7 of culture in BEC-SM. Other research groups earlier demonstrated *CD105* expression in epithelial cells (Schoonderwoerd et al., 2020).

According to the above results, it should be emphasized that even for experimental groups cultivated under the same medium conditions, different marker expression levels observed over time are a regular phenomenon in primary cultures (Fuchs et al., 2003).

## 5 Conclusions

**FIGURE 8:**
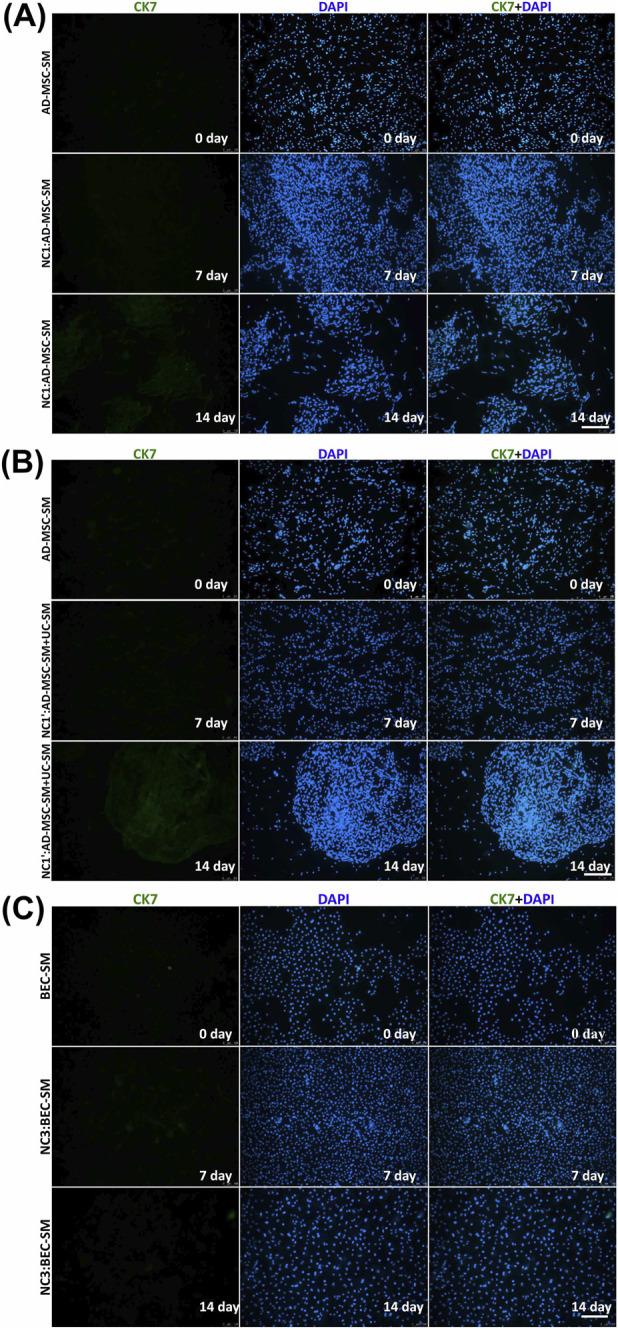
Representative fluorescence signals of CK7 and DAPI in **(A,B)** 0- day AD-MSC cultures in their SM (AD-MSC-SM); **(A)** 7- and 14-day NC1—AD-MSCs cultured in their SM, **(B)** 7- and 14-day N C1’ cultures—AD-MSCs cultured using a mixture of their SM and the SM for UCs, and **(C)** BECs cultured in SM (including cultures from day 0 and NC3 after 7 and 14 days of the experiment). Fluorescent microscopy, scale bar = 200 µm.

**FIGURE 9:**
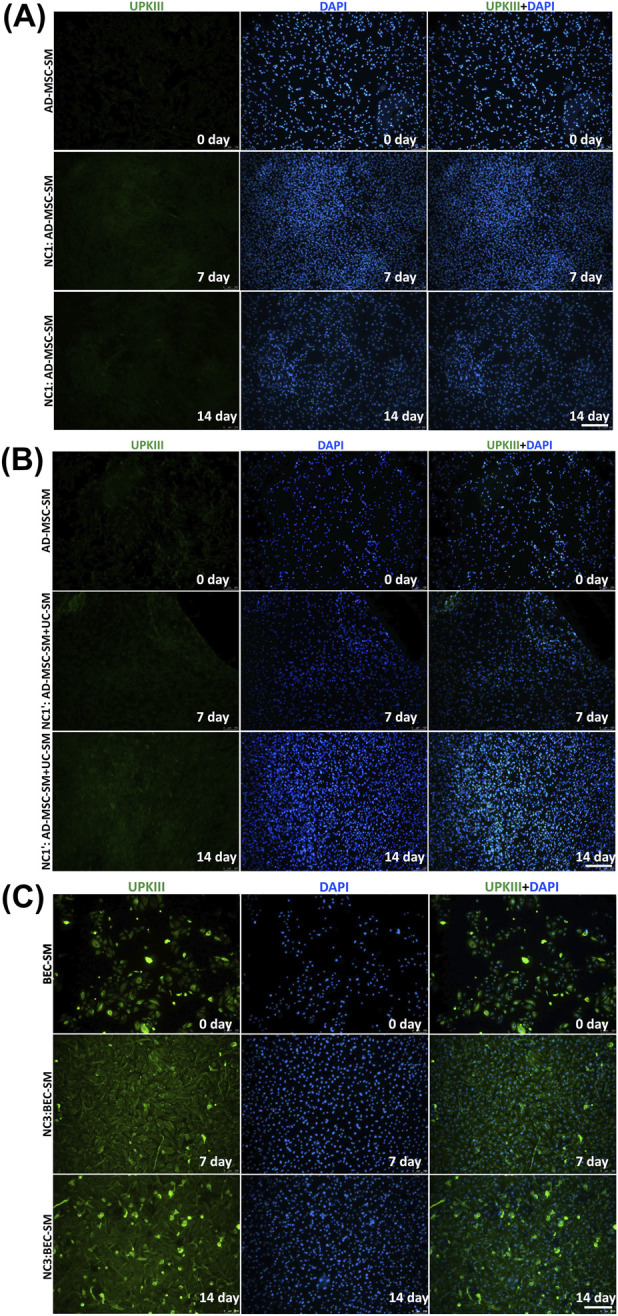
Representative fluorescence signals of UPKIII and DAPI in **(A,B)** day 0 AD-MSC cultures in their SM; **(A)** 7- and 14-day N C1 cultures, **(B)** 7- and 14-day NC1’ cultures, and **(C)** BECs cultured in their SM (including cultures from day “0″ and NC3 after 7 and 14 days of the experiment. BECs with positive CK7 expression are visible (green). Fluorescent microscopy, scale bar = 200 µm.

**FIGURE 10:**
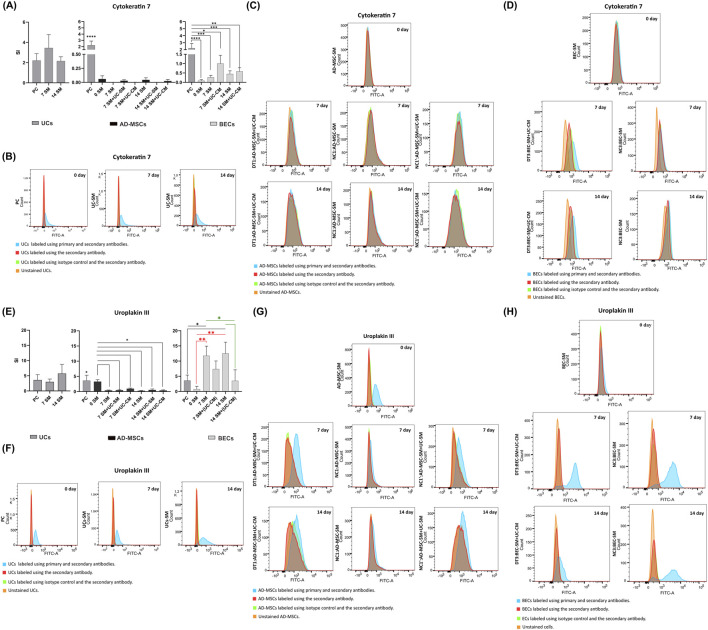
**(A)** SI after labeling with the antibody against CK7 of UC, AD-MSC, and BEC cultures that were cultivated from day 0 of experiment for 7 and 14 days using selected media (SM/the mixture (1:1) of SM with the unconditioned SM, typically used for UC culture (SM + UC-SM)/the mixture (1:1) of SM with UC-CM. Analyses were performed as multiple comparisons between all UC, AD-MSC, and BEC experimental groups and PC as mean ± standard deviation. *p < 0.05. **p < 0.01. ***p < 0.001.****p < 0.0001. Representative results of CK7 expression (a blue bar of the graph) in **(B)** UCs (PC and UC-SM), **(C)** AD-MSCs (AD-MSC-SM, DT1, NC1, and NC1’) and **(D)** BECs (BEC-SM, DT3, and NC3 from days 0, 7, and 14 of the experiment compared to controls (red, green, and orange bars of the graph). **(E)** SI after labeling with the antibody against UPKIII of UC, AD-MSC, and BEC cultures that were cultivated from day 0 of experiment for 7 and 14 days using selected media (SM, SM + UC-SM, and SM + UC-CM). Analyses were performed as multiple comparisons between all UC, AD-MSC, and BEC experimental groups and the PC as mean ± standard deviation. *p < 0.05. **p < 0.01. ***p < 0.001.****p < 0.0001. Representative results of UPKIII expression (a blue bar of the graph) in **(F)** UCs (PC and UC-SM), **(G)** AD-MSCs (AD-MSC-SM, DT1, NC1, and NC1’), and **(H)** BECs (BEC-SM, DT3, and NC3) from days 0, 7, and 14 of the experiment compared to controls (red, green, and orange bars of the graph).

In conclusion, we demonstrated that AD-MSCs and progenitor BECs differ in their potential to differentiate toward urothelial-like cells. pBECs have a higher potential for differentiation toward urothelial-like cells than AD-MSCs. UC-CM is the source of factors inducing such differentiation. pBECs express selected markers of highly specialized UCs, including *CK8*, *UPK1B*, and UPKIII, upon interaction with UC-CM. However, induction of the expression of UC early markers in this study was unsuccessful, which is an interesting result (Figures 5–10). Moreover, the results of this report indicated that the unconditioned medium generally used for UC culture (UC-SM) increased selected UC marker expression in pBECs. Its influence was positive but lower than UC-CM. These data prove that buccal epithelium can be an attractive cell source for tissue engineering purposes in reconstructive urology. BECs could be an alternative for obtaining UCs from external urinary bladder sources. Due to the importance of the urothelial layer and the potential advantages of its use in reconstructive urology, this study should be continued, including modifications to the protocol and more tests to assess the effectiveness of the differentiation process toward UCs.

## Data Availability

The raw data supporting the conclusions of this article will be made available by the authors, without undue reservation.

## Glossary

AD-MSCs: Adipose tissue-derived mesenchymal stromal/stem cells
BAM: Bladder acellular matrix
BECs: Buccal epithelial cells
bFGF: Basic fibroblast growth factor
BM-MSCs: Bone marrow mesenchymal stromal/stem cells
BPE: Bovine pituitary extract
BSA: Bovine serum albumin
CK: Cytokeratin
CM: Conditioned medium
DMEM: Dulbecco’s modified Eagle medium
DT: Differentiation trial
FBS: Fetal bovine serum
FC: Flow cytometry
H&E: Hematoxylin and eosin
IF: Immunofluorescence
ITG: Integrin
IHC: Immunohistochemistry
MSCs: Mesenchymal stromal/stem cells
NC: Negative control
OECs: Oral epithelial cells
pBECs: Progenitor buccal epithelial cells
PBS: Phosphate-buffered saline
PC: Positive control (UCs from the day when they reached subconfluence after the third passage (day 0))
pHPL: Pooled human platelet lysate
PPARγ1: Peroxisome proliferator-activated receptor gamma
RC: Radical cystectomy
RCF: Relative cell fluorescence
RT-qPCR: Real-Time quantitative polymerase chain reaction
SFM: Serum-free medium
SI: Staining index
SM: Standard medium
SMCs: Smooth muscle cells
TFs: Transcription factors
UC-CM: Conditioned medium from urothelial cell cultures
UCs: Urothelial cells
UPK: Uroplakin
ZO-1: Zonula occludens-1
