# Supplementary material for: Exploring the differentiation potential of adipose tissue-derived mesenchymal stromal/stem cells and progenitor buccal epithelial cells into urothelial cells
Source: Front Bioeng Biotechnol. 2026 Jan 12;13:1687541. doi: 10.3389/fbioe.2025.1687541 (PMC12832818; doi:10.3389/fbioe.2025.1687541)
Supplement: Supplementary file 1 [file DataSheet1.pdf]

Table S1 Analyzed markers and sequences used for primers construction.

Marker	Ensembl ID	Context Sequence
ACTβ (reference gene)	ENSSSCG00000007585	AAAAAGAACAATGTACAATCAAAGTCCTCGGCCACATTGTAAAACTTTGGGGGATGCTCCATCCAACCGACTGCTGTCACCTTCACCGTT CCAGTTTTTAAATCCTGAGTCAAGCGCCAAAAA
CD90	ENSSSCG00000015122	GCCAATGGTAGGGTTCATGGTGCCGGGGAGCTCAGTCCTGGACCTGGGTTCCGGTCCCTCGCCGCCGCAGACTGCTCCGGACACGCCGC CGCCTCCAGTTGCTGCACCCTGC
CD105	ENSSSCG00000005625	TTCCCCTTGGAAGTTGTGCCAGTCCAGATGGGGAGGCCGCTGGGAGCAGGTCCAGGTGGCTCTGGGCCGGACCTCTTCTGTTCTCGTGGA GGAGGTGCCTGTCAGCCTGGGCGGGCTG
CK8	ENSSSCG00000000252	GAGAGTCTTGATCTGCTCCTTCTCCTGGGTGCGCACGGCCTGGATGTTGGGGTCCACCTCCAGCTTAAGGGGGCTCAGCAGGCTCTGGTT CACTGTGACAGCTGTGATGCCTCCCAAGCCTGAGGGCCCA
CK13	ENSSSCG00000017445	TTTGCAAAAGGCAGGAAACTTTATTGAATGATCTTTCCTTTCTAGGAGAGAAGCCAAGAAACCAAAGGAGCCAGGTCAGGGAGAAGAG GAAGCAGACTCTGCGAAGAAGGGCAGGATGCACC
CK14	ENSSSCG00000017433	CCAAGGACTACAGCCCCTACTTCAAGACCATCGAGGACCTGCGGAACAAGATCCTCACGGCCACCGTGGACAACGCTAATGTCGTGCTG CAGATCGACAATGCCCGC
CK19	ENSSSCG00000017448	GGCAGAACCAGGAGTACCAGCATCTCATGGACATCAAGACGCGGCTGGAGCAGGAGATCGCCACCTACCGCAACCTGCTGGAGGGCCA GGACGCCAACTACAACAACCTGACCATCATCAAGGCCC
HPRT1 (reference gene)	ENSSSCG00000027175	TTTTCCAAATCCTCGGCATAATGATTAGGTATACAAAATAAATCTAGGTCATAACCTGGTTCGTCATCACTAATAGTTATGGAGGCTGGG AAGTCCAAGATCAAGGTAGCAGCAAAGTAGGTTTCATTTG
ITGβ1	ENSSSCG00000011101	AGTGGGACACTCTGGAGTCTCTACAACATGAACAGTGGCCTCATTGTTCCCATTCACCGAGTATGTGAAGTAGAACCAGCAATCATCAA CGTCCTTCTCCTTA
UPK1A	ENSSSCG00000002901	ACTTGGATTACCTGTTCACCAAGGGCTGCTTCGAACACATCGGCCACGCCATCGACAGCTACACATGGGGCATCTCGTGGTTTGGGTTTG CCATCCTGATGTGGACGCTCCCGGTG
UPK1B	ENSSSCG00000011904	GCAGCGGGTAGAGGCTATATTGGTCTGATACGAAGAAGATGCACTCTGCAGTCAGGGCGATGCCGCACATACCAATAATCACATTTCCA AAAACCAGCAGGCTCTGGAAGCAACGAACAGTGGAGTCATCTTTGGCCATC
UPK3A	ENSSSCG00000000015	GGTCCCCTTGGGGACGGCCTCCTGTGTGATCTGGGAGTCGTGGGTCATTTCCCCGTCCGCACCGCCCATGTCCATGAGGCTGAGGACGAT GGCGCCCGCAAAGCCGACGAGCAAGAAGAAGGGCAGG
UPK3B	ENSSSCG00000024796	GAGGGTCTGTGAGGCCTGAGCCTCCCTCTGTCCCTCTCTCTCATCCACCAGTGGCTGGGGCTGCAGTGAAGCCTTTAGAGATGCCTCTGC TGTCCCTGGACACTGGCCCTTTCTGAGGGTGGCTCTCACCGGCTGGAAGGTCAAACAGAAAGTAGACATGTTATGATCGTCTGGCCACCA CTTCTAAGCCCCTTGCACATAGATGGCTCATGGACTTATCCCCTCA
P63	ENSSSCG00000025592	GGAAGACCCCATCACAGGAAGACAGAGCGTGCTGGTACCTTACGAGCCACCTCAGGTTGGCACTGAATTCACGACAGTCTTGTACAATT TCATGTGTAACAGCAGTTGTGTTGGAGGAATGAACCGCCGTCC
RPL4 (reference gene)	ENSSSCG00000004945	TGATTCCTGGCCTGGCGAAGAATGGTGTTTCGGCGCATTGTCTTTGCATACGGGTTTAGCTTCAACATGATTCTCAGGTTTTTCAGTGGAT TCTTCTTCAGAACTCTACGATGAATCTTCTTGC
RPS18 (reference gene)	ENSSSCG00000001502	GCGCCACTTCTGGGGACTGCGTGTCCGAGGCCAGCACACCAAGACCACAGGCCGCCGTGGCCGCACCGTGGGTGTGTCCAAGAAGAAAT AAATGTGTCGGTGCTGCCTGTTAATAAATA
TBP (reference gene)	ENSSSCG00000022683	GAGTGGTGCCAGGCAAGGGTGCAGTCGTAAGAGTCTGTGAGTGGAAGAGCTGTGGAGTCTGGCCCGNTTGCTGCTCCTCCAAAATGGAT AG
